# Use and accuracy of decision support systems using artificial intelligence for tumor diseases: a systematic review and meta-analysis

**DOI:** 10.3389/fonc.2023.1224347

**Published:** 2023-10-04

**Authors:** Robert Oehring, Nikitha Ramasetti, Sharlyn Ng, Roland Roller, Philippe Thomas, Axel Winter, Max Maurer, Simon Moosburner, Nathanael Raschzok, Can Kamali, Johann Pratschke, Christian Benzing, Felix Krenzien

**Affiliations:** ^1^ Department of Surgery, Charité – Universitätsmedizin, Corporate Member of Freie Universität Berlin, Humboldt-Universität zu Berlin, and Berlin Institute of Health, Berlin, Germany; ^2^ Speech and Language Technology Lab, German Research Center for Artificial Intelligence (DFKI), Berlin, Germany; ^3^ Berlin Institute of Health (BIH), Berlin, Germany

**Keywords:** artificial intelligence, multidisciplinary team meetings, clinical decision support system, machine learning, concordance between CDSS and MDS

## Abstract

**Background:**

For therapy planning in cancer patients multidisciplinary team meetings (MDM) are mandatory. Due to the high number of cases being discussed and significant workload of clinicians, Clinical Decision Support System (CDSS) may improve the clinical workflow.

**Methods:**

This review and meta-analysis aims to provide an overview of the systems utilized and evaluate the correlation between a CDSS and MDM.

**Results:**

A total of 31 studies were identified for final analysis. Analysis of different cancers shows a concordance rate (CR) of 72.7% for stage I-II and 73.4% for III-IV. For breast carcinoma, CR for stage I-II was 72.8% and for III-IV 84.1%, P≤ 0.00001. CR for colorectal carcinoma is 63% for stage I-II and 67% for III-IV, for gastric carcinoma 55% and 45%, and for lung carcinoma 85% and 83% respectively, all P>0.05. Analysis of SCLC and NSCLC yields a CR of 94,3% and 82,7%, P=0.004 and for adenocarcinoma and squamous cell carcinoma in lung cancer a CR of 90% and 86%, P=0.02.

**Conclusion:**

CDSS has already been implemented in clinical practice, and while the findings suggest that its use is feasible for some cancers, further research is needed to fully evaluate its effectiveness.

## 1 Introduction

Cancer is one of the leading causes of death worldwide (1). In 2020, 10 million people worldwide died from cancer (2). Interdisciplinary tumor boards or multidisciplinary team meetings (MDMs) are the backbone in treatment planning for patients with tumor disease (3). MDMs are usually held on a weekly basis, with the goal of finding the best treatment based on current guidelines and medical evidence. Indeed, medical guidelines strongly recommend discussing patients in MDMs prior to the actual treatment (4).

The goal of MDMs is to weigh potential treatment options based on available patient data and radiological exams. A complete set of the required patient data including performance status, tumor stage and co-morbidities is required for effective decision-making (5). In most countries, data are currently entered manually into simple online forms such as the Giessen Tumor Documentation System (GTDS) in preparation for MDMs (6). Administrative and procedural difficulties in retrieving patient information are not uncommon, usually due to missing pathology and radiology results or incomplete information on referral forms from other medical institutions (7). Thus, missing data can lead to delays in diagnosis and treatment (8). Moreover, excessive workload and time pressure adversely affect MDMs (9), which can in turn lead to unstructured case discussions and variability in the quality of decision-making.

**Figure 1:**
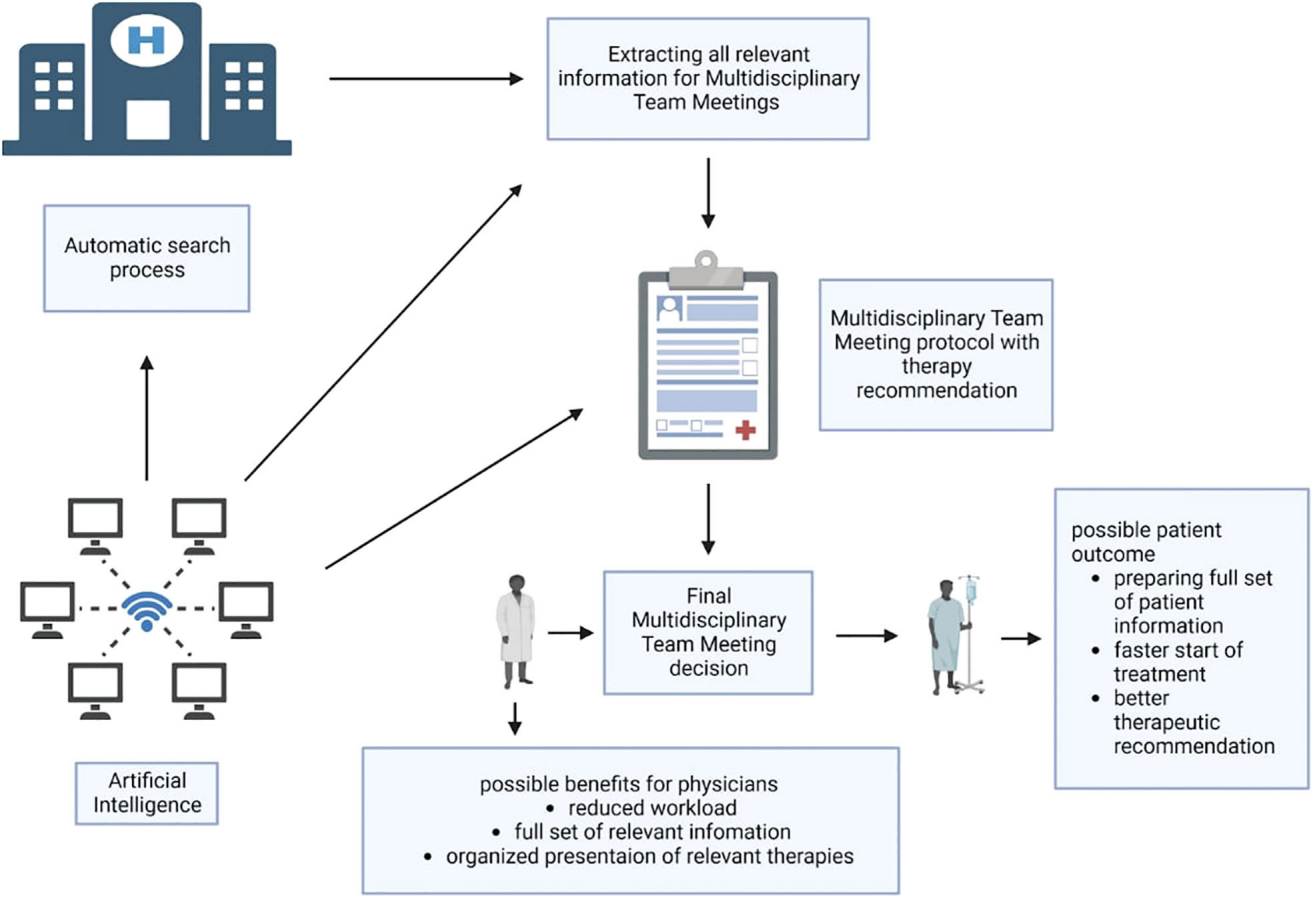
Possible workflow of AI supporting MDMs. An automated program using artificial intelligence (machine learning, natural language processing) runs in the background of the hospital information system and can extract relevant data for MDM from the system. Afterwards, the tumor board protocol can be automatically prepared and filled out with all relevant patient data in preparation for the MDM. At the same time, the program could provide treatment suggestions based on the available data and support these with existing guidelines or studies. Based on this, the physicians in the MDM can then make the therapy decision. In the end, both physicians and patients could benefit. Created with BioRender.com.

To overcome the current problems in conventional MDMs, automated processes and decision support systems might help. There is increasing research on AI and machine learning (ML) techniques applied in MDM (**Figure 1**). In recent times, artificial intelligence (AI) is viewed as a branch of engineering that implements novel concepts and solutions to resolve complex challenges. With rapid advancements in technology, computers may someday be as intelligent as humans (10). Today, the natural language processing (NLP) model ChatGPT can hold conversations and produce meaningful text such as e-mail or essay writing when given prompts via a dialogue format (11). In medicine, AI can be divided into two main branches: virtual and physical (10). ML is an area of AI that aims to process large amounts of qualitative information to identify patterns of relevant information.

The objective of this review is to provide an overview and systematic analysis of the current usage and accuracy of AI-based decision support systems in MDM. Specifically, the review will focus on studies that evaluate the consistency between AI-based decision support systems and MDM decisions.

## 2 Methods

This review was conducted according to the PRISMA guidelines for systematic reviews (12) and was registered with the International Prospective Register of Systematic Reviews (PROPSPERO ID: 411462).

### 2.1 Eligibility criteria

The studies considered for this review met the following criteria:

- The studies verified the consistency of AI-based systems in MDM, regardless of cancer type.
- The studies thoroughly compared the consistency of treatment regimens established by AI and MDM, specifically the correspondence between AI decisions and those made by a multidisciplinary team or using established standards such as guidelines.
- Only studies with adult patients aged 18 and above were included.
- The studies were available in full text and written in English.
- Only retrospective and prospective studies were considered.

### 2.2 Exclusion criteria

· The study does not fulfill the inclusion criteria.

· The article is a systematic review or meta-analysis.

### 2.3 Literature search methodology

**Figure 2:**
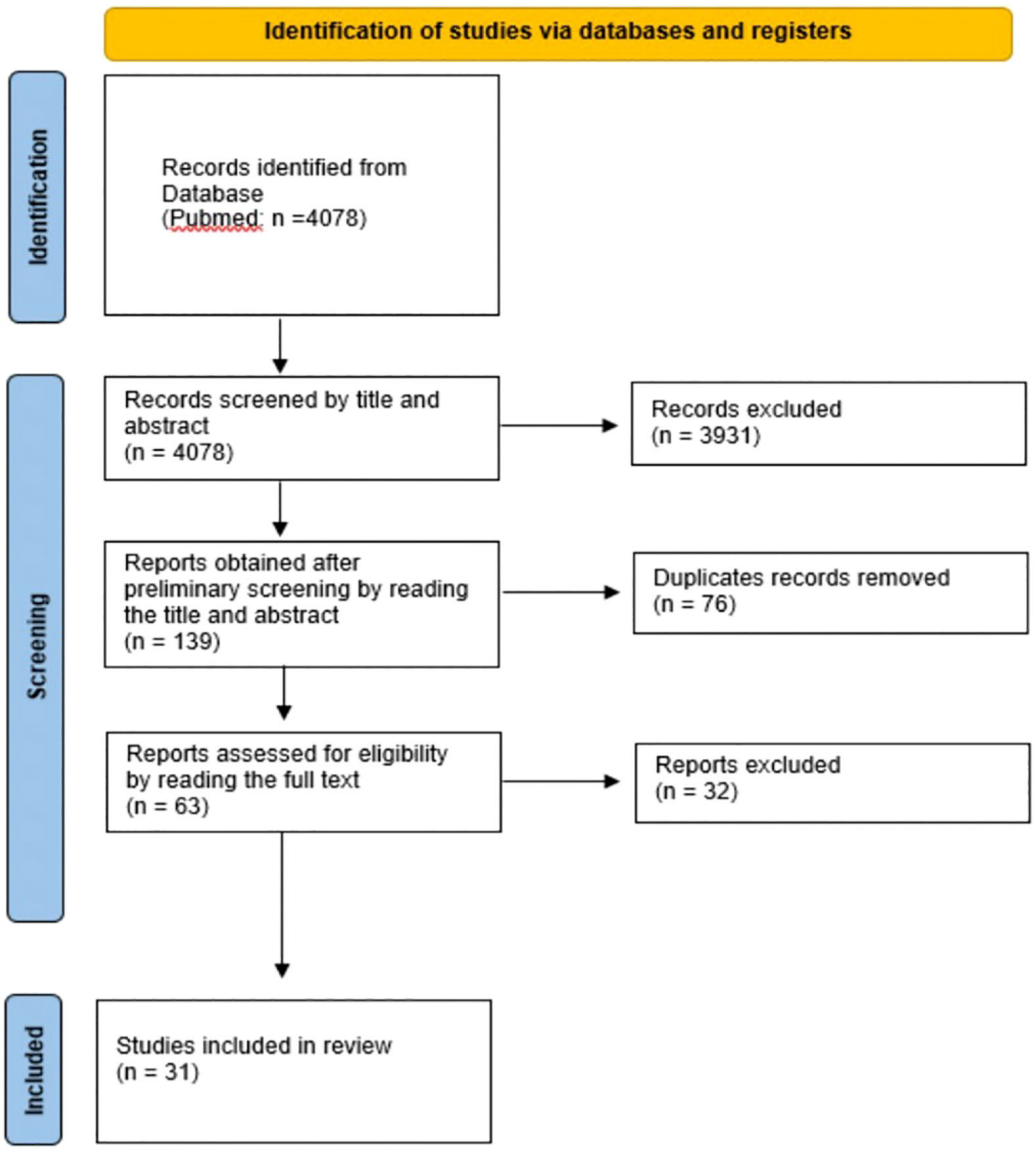
Flow diagram of the study selection process. This figure was designed according to the PRISMA-Statement (13).

The present review was conducted according to the PRISMA guideline for systematic reviews (**Figure 2**) (13). The literature research on Pubmed (MEDLINE) was carried out until November 2022 using MeSH keyword search. The search terms were the following: (machine learning) AND (tumor board); (machine learning) AND (multidisciplinary team meetings); (machine learning) AND (multidisciplinary cancer teams); (artificial intelligence) AND (multidisciplinary cancer teams); (artificial intelligence) AND (multidisciplinary team meetings); (artificial intelligence) AND (tumor board); IBM Watson for Oncology; (machine learning) AND (multidisciplinary team); Watson for Oncology; (artificial intelligence) AND (multidisciplinary team); (clinical decision support system) AND (multidisciplinary team meetings); (clinical decision support system) AND (multidisciplinary team); (clinical decision support system) AND (multidisciplinary cancer teams); (clinical decision support system) AND (tumor board).

For the search terms “Watson for Oncology” and “IBM Watson for Oncology”, the search was limited to literature from 2015 onwards, because commercial use of Watson for Oncology began in 2015 (14). For all other search terms, no time limit was set. In total, 4078 records were identified through database searching. Preliminary screening of titles, abstracts and duplicates yielded 139 articles. The aim of the paper was to include studies that focused on CDSS and then review the concordance. However, the very general selection of search terms resulted in in a large list of papers that deal with AI in oncology but did not cover any CDSS. Indeed, this was recognizable in most cases by title and abstract. Note, a decent amount of duplicates have been removed as well (n = 823). After the initial selection process, the articles were read in full and care was taken to review both treatment recommendations and concordance between CDSS and MDM. Excluded were articles that compared CDSS to a guideline, investigated how CDSS influences the actions of MDMs, articles that investigated the acceptance of CDSS by physicians or patients, or articles in which CDSS provided a prognosis or could decide on possible inclusion in a trial. Meta-analyses or reviews on the topic were also excluded. Finally, after independent assessment of full text articles by two different researchers (RO, SN), 31 articles were included. No separate checks on study quality like patient selection or study population were done. Studies were included when they had performed an analyzation of concordance rate between MDM and CDSS. If there was disagreement on this, an additional independent arbitrator (FK) was consulted for further resolution.

### 2.4 Statistical analysis

Review Manager (RevMan) 5.4.1 (The Cochrane Collaboration, 2020) software was utilized to conduct a comprehensive analysis of the extracted data. To enhance the clarity and ease of interpretation of the results, forest plots were generated. The primary objective was to assess the level of agreement between treatment decisions made by WFO and MDT for various cancer types. The data was analyzed dichotomously, and odds ratios (ORs) with corresponding 95% confidence intervals were calculated for each variable (stage, histology type, etc.) Heterogeneity among the studies was evaluated using the I^2^ test. I^2 ^> 50% indicated considerable heterogeneity, whereas no heterogeneity was present in the absence of these conditions. P < 0.05 was considered significant. If the data provided could not be meta-analyzed, only descriptive analysis was done. Because not all studies could be included in the meta-analysis due to the unavailability data, an additional descriptive analysis was performed.

## 3 Results

### 3.1 Study characteristics

Most of the studies which matched the review criteria used Watson for Oncology (WFO). Twenty-three of the 31 studies were on WFO and concordance (**Table 1**). Other Clinical Decision Support Systems (CDSSs) included were OncoDoc (15), Lung Cancer Assistant (LCA) (17) and the Multidisciplinary meeting Assistant or Treatment sElector (MATE) (16). Two studies using a decision tree model based on Dutch guidelines were included (41). In addition to the CDSSs mentioned above, there were two prototype decision tree models created by the working group Andrew et al. and Lin et al. that conducted a concordance study (18, 42).

**Table 1:** Overview of studies on decision support systems using artificial intelligence for tumor diseases; n refers to the actual number analyzed.

Team	Year	Journal	n	Tumor Entity	Decision instance that is being compared to AI
Séroussi B et al. (15)	2007	AMIA Annu Symp Proc.	241	breast	MDM
Patkar V et al. (16)	2012	BMJ Open	1056	breast	MDM
Sesen MB et al. (17)	2014	J R Soc Interface	4020	lung	Comparison between Patient treatment from English National Lung Cancer Audit Database and decision of LCA
Lin FP et al. (18)	2016	BMC Cancer.	1065	breast	MDM
Somashekhar SP et al. (19)	2018	Ann Oncol.	638	breast	MDM
Kim YY et al. (20)	2018	PLoS One	95	breast	Real clinical practice
Zhou N et al. (21)	2019	Oncologist	362	lung, breast, gastric, colon, rectal, cervical, ovarial	Physicians at cancer center
Liu C et al. (22)	2018	J Med Internet Res	149	lung	Actual treatment
Lee WS et al. (23)	2018	JCO Clin Cancer Inform.	656	colon	MDM
Choi YI et al. (24)	2019	Can J Gastroenterol Hepatol.	65	gastric	MDM
Kim EJ et al. (25)	2019	PLoS One	69	colorectal	MDM
Kim M et al. (26)	2019	Cancer	207	thyroid	physician’s recommendation after thyroidectomy for radioactive iodine therapy
McNamara DM et al. (27)	2019	Cancer Med	223	breast	oncologist
Zhang W et al. (28)	2020	World J Surg	234	HCC	surgeons; only patients who received surgery
Tian Y et al. (29)	2020	J Med Internet Res.	235	gastric	MDM
Yao S et al. (30)	2020	Thorac Cancer	165	lung	physicians
You HS et al. (31)	2020	Cancer Manag Res	310	lung	MDM
Zou FW et al. (32)	2020	Front Genet	246	cervical	MDM
Yu SH et al. (33)	2021	World J Urol	201	prostate	MDM
Zhao X et al. (34)	2020	Jpn J Clin Oncol	302	breast	MDM
Kim MS et al. (35)	2020	Cancer Res.	405	lung	MDM
Xu F et al. (36)	2020	JCO Clin Cancer Inform	1977	breast	oncologist
Mao C et al. (37)	2020	Front Oncol	175	colorectal	MDM
Suwanvecho S et al. (38)	2021	J Am Med Inform Assoc	313	breast, colon, lung, rectal	Physician decision (not specified if it was part of MDM)
Aikemu B et al. (39)	2021	Front Oncol.	250	colorectal	MDM
Yun HJ et al. (40)	2021	Front Endocrinol	50	thyroid	recommended treatment according to the Korean Thyroid Endocrine Surgery Association guidelines (Kates)
Keikes L et al. (41)	2021	Int J Qual Health Care	127	colorectal	MDM
Andrew TW et al. (42)	2022	Br J Cancer	304	basal cell	MDM
Pan H et al. (43)	2019	Transl Cancer Res.	1301	breast	oncologist
Ebben KCWJ et al. (44)	2022	Int J Qual Health Care	296	breast, prostate, colorectal	MDM
Liu Y et al. (45)	2022	Clin Exp Med.	463	breast	Actual treatment

Across all studies, a total of 16,472 participants were included. The number of included subjects varied greatly within the included studies. Five studies had a very small number of cases (< 100) (20, 24, 25, 27, 40) while the other studies had a relatively large number of included cases(> 1000) (16–18, 36, 43). Three studies examined multiple tumor entities, and included only a small number of participants in the subgroups (21, 38, 44).

Thirteen studies of breast cancer were conducted, involving a total of 7786 subjects (15, 16, 18–21, 27, 34, 36, 38, 43–45). The number of participants per study varied widely, ranging from 55 (27) to 1,977 (36). The most common treatment decisions reviewed in this study were for breast cancer, with the MDM and CDSS evaluated in multiple studies. Colorectal cancer was the subject of eight studies (21, 23, 25, 37–39, 41, 44), followed by lung cancer with seven studies (17, 21, 22, 30, 31, 35, 38). Gastric cancer was reviewed in three studies (21, 24, 29), while cervical (21, 32) and prostate cancers (33, 44) were each the focus of two studies. Thyroid cancer was examined in two studies (26, 40), and there was one study each for ovarian cancer (21), basal cell carcinoma (42), and hepatocellular carcinoma (HCC) (28).

Of the analyses evaluating the concordance rate between therapy decisions and CDSS, the majority were retrospective. Only three analyses were prospective (15, 16, 44).

### 3.2 Clinical decision support systems

As seen in **Table 1**, there are several AI-based CDSSs used regularly in clinical oncology. The most common is Watson for Oncology (WFO); its use is widespread in the US and in Asia. Other systems like OncoDoc, LCA or other decision tree-based CDSSs are often prototypes which are only used at a single hospital, region or country. The CDSSs that were reviewed for concordance with treatment decisions are appointed below.

#### 3.2.1 Watson for oncology

WFO is an AI CDSS developed by IBM Corporation (USA) in cooperation with oncologists from Memorial Sloan Kettering Cancer Center (USA) (46). For supported cases, the treatment recommendations provided by WFO fall into three possible categories: *´Recommended´*, *´For consideration´* and *´Not recommended´* (14).

#### 3.2.2 OncoDoc

OncoDoc is a CDSS based on clinical practice guidelines (CPGs) that allows physician discretion in the decision-making process. CPGs are organized in decision trees. Decision parameters are dynamically instantiated by the physicians. It was developed in collaboration with the medical oncology department of the Pitié-Salpêtrière Hospital (France) and has first been applied to the treatment of breast cancer (47).

#### 3.2.3 Lung cancer assistant

LCA is a CDSS prototype designed in the United Kingdom. Probabilistic and guideline rule-based decision support are used to aid clinicians’ decision-making in lung cancer MDMs (17).

#### 3.2.4 Oncoguide

Oncoguide is an open access, interactive decision support software developed in the Netherlands with the help of a multidisciplinary team. The Dutch CPGs for colorectal cancer were converted into decision trees and then validated with patient data. Supporting information from the CPGs, such as scientific evidence for specific treatment decisions, are presented with the recommendations (41, 44).

#### 3.2.5 MATE

MATE (Multidisciplinary meeting Assistant and Treatment sElector) is a CDSS developed in the United Kingdom and used in breast cancer MDMs. It requires manual input of patient data by a physician, assesses patient eligibility for clinical trials and presents ranked recommendations together with supporting evidence (16).

### 3.3 Results of meta-analysis and concordance rate

**Figure 3:**
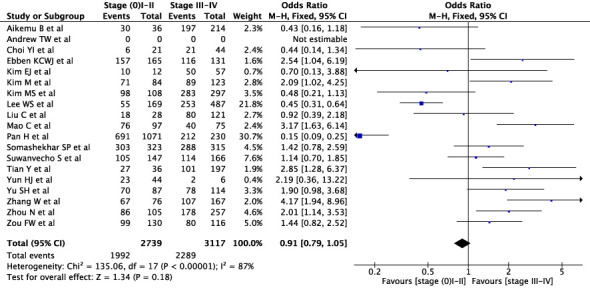
Overall concordance of various cancers in stages I–II and III-IV.

**Figure 4:**
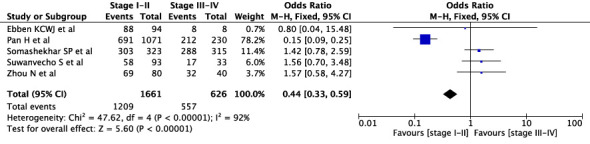
Overall concordance in breast cancer in stages I-II and III-IV.

First, we conducted an overall meta-analysis of patients with different cancer stages (see **Figure 3**). In studies concerning WFO, treatment was deemed concordant if it was categorized as *‘Recommended’* or *‘For consideration’*. A total of 18 studies were included in the analysis. The results showed a concordance rate of 72.7% (1992/2739) for stages I-II and 73.4% (2289/3117) for stages III-IV across various carcinomas, although this difference was not statistically significant (P=0.18). However, the meta-analysis revealed significant statistical heterogeneity (I^2^ = 88%) across different cancer stages. As a result, we conducted a subgroup meta-analysis to examine specific cancer types and stages. In the case of breast cancer, five studies were included in the analysis (see **Figure 4**), revealing a concordance rate of 72.8% (1209/1661) for stages I-II and 84.1% (557/662) for stages III-IV, P≤ 0.00001.

**Figure 5:**
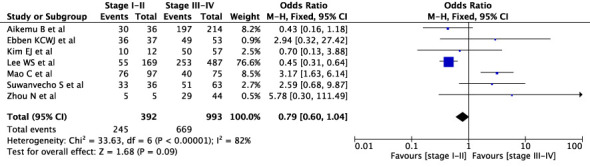
Overall concordance in colorectal cancer in stages I-II and III-IV.

**Figure 6:**
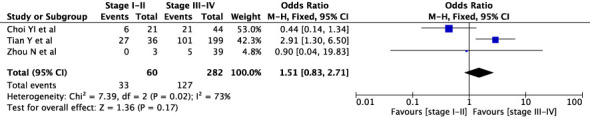
Overall concordance in gastric cancer in stages I-II and III-IV.

**Figure 7:**
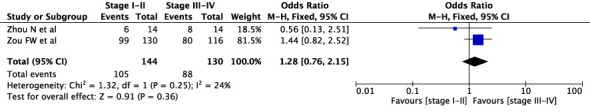
Overall concordance in cervical cancer in stages I-II and III-IV.

**Figure 8:**
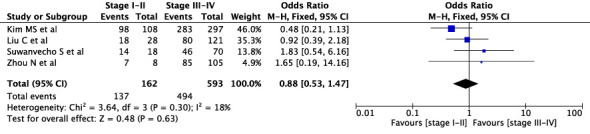
Overall concordance in lung cancer in stages I-II and III-IV.

**Figure 9:**
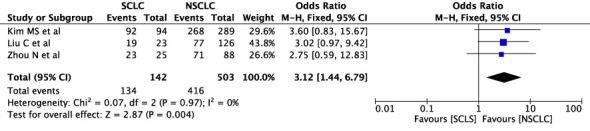
Overall concordance in different lung cancer types for SCLC and NSCLC.

**Figure 10:**
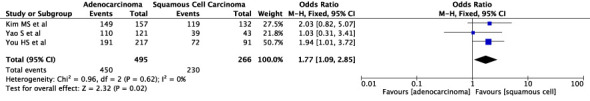
Overall concordance in NSCLC for histopathology type for adenocarcinoma and squamous cell carcinoma.

**Figure 11:**
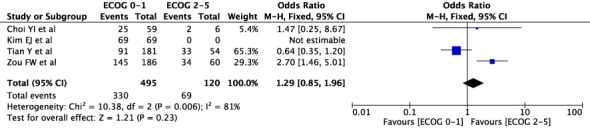
Overall concordance for ECOG 0-1 and 2-5.

The concordance rates for different cancer types and stages were as follows: for colorectal carcinoma (**Figure 5**), 63% (245/392) for stages I-II and 67% (669/993) for stages III-IV; for gastric carcinoma (**Figure 6**), 55% (33/60) for stages I-II and 45% (127/282) for stages III-IV; for cervical cancer (**Figure 7**) for stages I-II 73% (105/144) and 68% (88/130) for stage III-IV; and for lung carcinoma (**Figure 8**), 85% (137/162) for stages I-II and 83% (494/593) for stages III-IV. However, none of these differences were statistically significant (P>0.05). In addition, we analyzed different types of lung cancer, including SCLC and NSCLC, in three studies (**Figure 9**). The results showed a concordance of 94.3% (134/142) for SCLC and 82.7% (416/503) for NSCLC, with a statistically significant difference (P=0.004). Analysis of histopathology subtypes in lung cancer revealed a concordance rate of 90% (450/495) for adenocarcinoma and 86% (230/266) for squamous cell carcinoma (**Figure 10**), with a statistically significant difference (P=0.02). For ECOG 0-1, the concordance rate was 66.6% (330/495), while for ECOG 2-5 (**Figure 11**), it was 58% (69/120), although this difference was not statistically significant (P=0.23).

Breast cancer has been analyzed by various CDSSs, showing generally high concordance. In the study of Somashekhar et al., the overall concordance rate between WFO and MDM is near 93% being at the ´Recommend´ level 62% and the `For consideration´ level 31% (19). Across the different stages, the concordance is above 80% (19), which is the same in the study of Zhou N et al. (21) As for the other CDSSs, there is also a high concordance rate of 93,4%, 93,2% and 85,3% using OncoDoc2, MATE and decision clinical tree system based on Oncoguide respectively (15, 16, 44). McNamara et al. conducted a study to analyze the concordance of WFO with decisions made by oncology experts and its impact on decisions made by newcomers to oncology. In breast cancer, the overall concordance rate among experts was found to be 87.9%. Novice oncologists had a concordance rate of 75.5% without the use of WFO, which improved to 95.3% with WFO (27).

In a study by Zhao et al., concordance rates between MDM and WFO were found to be only 77% for the adjuvant treatment group and 27.5% for the metastatic group (34). Xu et al. conducted an interesting study on the influence of WFO on treatment decisions, which showed that treatment decisions changed in only 5% of cases after reviewing WFO recommended treatment options for patients (36). However, there were also studies on breast cancer with low concordance rates, such as a study by Suwanvecho et al., which found a concordance rate of only 59.3% (38). In a study by Pan et al., the overall concordance rate was only 69.4%. Interestingly, the concordance rate was worse in the adjuvant chemotherapy group, whereas in the neoadjuvant chemotherapy group, the overall concordance rate was 96.7% (43).

Studies evaluating the use of WFO in patients with colorectal carcinoma have shown highly variable results. Some studies, including Zhou et al., Lee et al., and Mao et al., reported low overall concordance rates of 64%, 48.9%, and 66.9%, respectively, for colorectal cancer (21, 23, 37). However, other studies, such as Kim et al. and Aikemu et al., reported good agreement rates with overall concordance of 87% and 91%, respectively, for colorectal cancer (25, 39). Additionally, two studies that did not use WFO as a clinical decision support system also reported good overall concordance rates above 80% (41, 44).

Several studies have been conducted on lung cancer and WFO. Kim et al. achieved a high concordance rate of 92.4% (35). Zhou et al. showed an overall concordance rate of 83%, 92% for SCLS and 80% for NSCLC (21). In contrast, Liu et al. reported only an overall concordance rate of 65.8%, but also achieved 83% for SCLS but only 61.1% for NSCLC (22). Two of the studies discussed in this paper were conducted just for NSCLC, You et al. recorded a high overall concordance rate of 85.16% compared to the other studies, and Yao et al. achieved 73.3%, which was higher than the work of Liu et al. (30, 31) Sesen et al. used the LCA system, in which the rule-based decision support of the LCA guideline achieved an exact concordance rate of 0.57 with the recorded treatments. For the probabilistic LCA decision aid, the result was worse, with 0.27 and 0.76 for the exact and partial concordance rates, respectively. In this study, MDM was not performed, but patient treatment from the English National Lung Cancer Audit Database was compared with the LCA decision (17).

The overall concordance rate for gastric cancer was low at 54.5% by Tian et al. (32) In a study by Choi et al, concordance at the recommended level was also low at 41.5%, but higher at the recommendation level at 87.5%. For various stages and low ECOG scores, consensus was also low (24).

Two cervical cancer studies were found for this review. In both studies, overall agreement was below 75% with 64% and 72.8%, respectively (21, 32).

Yu et al. showed an overall concordance of 73,6% for prostate cancer. Looking at the different stages there was a higher concordance for lower stages (33). Ebben et al. showed in there study a similar overall concordance (78,8%) but using a different CDSS (44).

For thyroid cancer the results are diverse. The study of Yun et al. showed only an overall recommendation of 48% (40) in contrast to 77% overall concordance shown in the study by Kim et al. (26).

For ovarian cancer Zhou et al. showed a concordance rate above 90% overall and for stages as well (21).

Andrew et al. did a study on a Machine-learning algorithm to predict multidisciplinary team treatment recommendations in the management of basal cell carcinoma (42). They stated that the choice of conventional treatment (surgical excision or radiotherapy) by the MDT could be reliably predicted based on the patient’s age, tumor phenotype and lesion size. The algorithm reliably predicted the MDT decision outcome of 45.1% of nasal Basal cell cancer (42).

Zhang et al. conducted a study on hepatocellular carcinoma (HCC), where only surgically treated patients were included. The study aimed to compare the concordance between the decision made by WFO and the decision made by surgeons regarding the need for surgery, without comparing with MDM. The overall concordance rate was found to be 72%. In subgroup analyses, concordance varied from 66% for major hepatectomy to 88% for BCLC stage 0-A, indicating a higher level of agreement in less complex cases (28) (**Table 2**).

**Table 2:** Concordance rate between AI system and MDM; Not every subgroup analysis has been included in the table.

Title	Team	Tumor entity	AI system used	Concordance rate between AI system and MDM														
Supporting multidisciplinary staff meetings for guideline-based breast cancer management: a study with OncoDoc2	Séroussi B et al.	Breast	OncoDoc2	-Without OncoDoc2 72,9% -With OncoDoc2 93,4%														
Using computerized decision support to improve compliance of cancer multidisciplinary meetings with evidence-based guidance	Patkar V et al.	Breast	MATE	Overall: 93,2%														
Lung Cancer Assistant: a hybrid clinical decision support application for lung cancer care	Sesen MB et al.	Lung	LCA	Overall concordance rate - LCA guideline rule-based decision support achieved an exact concordance rate of 0.57 with the recorded treatments 0.79 when partial matches were included -LCA probabilistic decision support 0.27 exact and 0.76 for partial concordance														
Computational prediction of multidisciplinary team decision-making for adjuvant breast cancer drug therapies: a machine learning approach	Lin FP et al.	Breast	Different ML models Bootstrap-aggregated decision trees	MDT Recommendation				Best ML modell Sens/Spec			ESMO Guidelines Sens/Spec			NCCN Guidelines Sens/Spec				
				Chemotherapy - Aggressive 60% - Conservative 35%				- 0.93/0.89 - 0.86/0.95			- *0.55/0.78* *- 0.60/0.82*			- 0.97/*0.12* - 0.82/*0.71*				
				Endocrine - Aggressive 91% - Conservative 79%				- 0.98/0.85 - 0.97/0.65			- 0.98/0.81 - 0.99/*0.36*			- 0.97/0.75 - 0.96/0.50				
				Trastuzumab - Aggressive 20% - Conservative 19%				- 0.98/0.99 - 0.95/0.99			- 0.97/0.97 - 0.97/0.96			- 0.97/0.98 - 0.92/0.98				
Watson for Oncology and breast cancer treatment recommendations: agreement with an expert multidisciplinary tumor board	Somashekhar SP et al.	Breast	WFO	Overall				By stage						Receptor status				
				Total 93% Recommended 62% for consideration 31%				Stage I: Total 80%; Recommended 51% for consideration 29%						HR+ Non metastatic Total 95%; Recommended 59% for consideration 36% metastatic Total 75%; Recommended 55% for consideration 20%				
								Stage II: Total 97%; Recommended 64% for consideration 33%						HER2/neu+ Non metastatic Total 94%; Recommended 56% for consideration 38% metastatic Total 98%; Recommended 64% for consideration 34%				
								Stage III: Total 95%; Recommended 64% for consideration 31%						Triple – Non metastatic Total 94%; Recommended 74% for consideration 20% metastatic Total 85%; Recommended 65% for consideration 20%				
								Stage IV: Total 86%; Recommended 61% for consideration 25%										
Gene expression assay and Watson for Oncology for optimization of treatment in ER-positive, HER2-negative breast cancer	Kim YY et al.	Breast	WFO	Concordant therapeutic recommendations between real clinical practice and WFO -without Gene Expression Assay were obtained for 23.2% of the patient group -with Gene Expression Assay Sensitivity, specificity, positive predictive and negative predictive values of WFO with Gene Expression Assay were 100%, 80%, 61% and 100%														
Concordance Study Between IBM Watson for Oncology and Clinical Practice for Patients with Cancer in China	Zhou N et al.	Lung, Breast, Gastric, Colon, Rectal, Cervical, Ovarial	WFO	Lung - Overall 83% - SCLC 92% - NSCLC 80% - Tumor stage: stage II 87.5% Stage III 75.8% stage IV 84.6%	Breast - Overall 82% - Histology: Luminal A 63%; Luminal B 87%; Triple – 79% - stage: Stage II 86%; Stage III 80%				Gastric - Overall 12% - Stage: Stage II 0% Stage III 19% Stage IV 6%		Colon - Overall 64% - stage: Stage II 100% Stage III 67& Stage IV 50%		Rectal - Overall: 74% - Stage: Stage II 100% Stage III 75% Stage IV 73%	Cervical - Overall: 64% - Stage: Stage II 33% Stage III 100% Stage IV 83%		Ovarian - Overall: 96% - Stage: Stage II 100% Stage III 100% Stage IV 93%		
Using Artificial Intelligence (Watson for Oncology) for Treatment Recommendations Amongst Chinese Patients with Lung Cancer: Feasibility Study	Liu C et al.	Lung	WFO	Overall					Type		Stage			Non-metastatic				
				Total 65,8% Recommended 42,3% For Consideration 25,5%					- SCLS Total 83% Recommended 78,3% For Consideration 4,3%		- Stage I Total 83% Recommended 33,3% For Consideration 50%			Total 49% Recommended 23,2% For Consideration 25,6%				
									- NSCLC Total 61,1% Recommended 33% For Consideration 27,8%		- Stage II Total 59% Recommended 9,1% For Consideration 50%							
											- Stage III Total 42% Recommended 27,1% For Consideration 15,1%							
											- Stage IV Total 89% Recommended 69,3% For Consideration 19,4%							
Assessing Concordance With Watson for Oncology, a Cognitive Computing Decision Support System for Colon Cancer Treatment in Korea	Lee WS et al.	Colon	WFO	Overall							Stage							
				Total: 48,9%							Stage II: 32,5%							
											Stage III: 58,8%							
											Stage IV: 35,9%							
Concordance Rate between Clinicians and Watson for Oncology among Patients with Advanced Gastric Cancer: Early, Real-World Experience in Korea	Choi YI et al.	Gastric	WFO	Overall					Stage (Recommended)					ECOG				
				Recommended 41,5%					Stage II: 22,2%					ECOG 0, 1: 92,6%				
				For consideration 87,7%					Stage III: 55,6%					ECOG 2, 3: 7,4%				
									Stage IV: 22,2%									
Early experience with Watson for oncology in Korean patients with colorectal cancer	Kim EJ et al.	Colorectal	WFO	MDM - Total 87% (Recommended 46,4%; For Consideration 40,6%)							NCCN guidelines - Total 92,8% (Recommended 88,4%; For Consideration 4,4%)							
Concordance in postsurgical radioactive iodine therapy recommendations between Watson for Oncology and clinical practice in patients with differentiated thyroid carcinoma	Kim M et al.	Thyroid	WFO	Overall					ATA Risk Classification		TNM (7^th^ edition)			TNM (8^th^ edition)				
				Total: 77%					Low: 91%		Stage I: 84%			Stage I: 76%				
									Intermediate: 61%		Stage II: 100%			Stage II: 82%				
									High: 100%		Stage III: 65%							
											Stage IV: 100%							
Differential impact of cognitive computing augmented by real world evidence on novice and expert oncologists	McNamara DM et al.	Breast	WFO	Breast Cancer Experts - Total 87,9 % - Recommended 78,5% - For Consideration 9,4%							novice oncologists - Without WFO/Cota RWE 75.5% recommended/for consideration - improved to 95.3% with WFO/Cota RWE - changed decisions in 39% cases with WFO/Cota RWE							
Concordance Study in Hepatectomy Recommendations Between Watson for Oncology and Clinical Practice for Patients with Hepatocellular Carcinoma in China	Zhang W et al.	HCC	WFO	Overall			Number of tumors		Extension of hepatectomy		type of resection			BCLC stage				
				Total: 72%			Solitary: 86%		Major: 66%		Anatomical: 72%			Stage 0-A: 88%				
							Multiple: 67%		Minor: 81%		Nonanatomical: 71%			Stage B-C: 64%				
Concordance Between Watson for Oncology and a Multidisciplinary Clinical Decision-Making Team for Gastric Cancer and the Prognostic Implications: Retrospective Study	Tian Y et al.	Gastric	WFO	Overall					Stage					HER2 status				
				- Total: 54, 5% - Recommended: 43% - For Consideration: 11,5%					Stage I: 77%					HER2-: 56,1%				
									Stage II: 74%					HER2+: 39%				
									Stage III: 52,5%									
									Stage IV: 48%									
Real world study for the concordance between IBM Watson for Oncology and clinical practice in advanced non-small cell lung cancer patients at a lung cancer center in China	Yao S et al.	Lung (NSCLC)	WFO	Overall			Sex		Smoking			ECOG		Stage				Pathology
				73,3%			Male: 93,6%		Yes: 91,4%			0: 92%		III: 77,8%				Squamous: 90,7%
							Female: 85,7%		Non: 90,5%			1: 90,4%		IV: 93,5%				Adeno: 90,9%
Concordance of Treatment Recommendations for Metastatic Non-Small-Cell Lung Cancer Between Watson for Oncology System and Medical Team	You HS et al.	Lung (NSCLC)	WFO	Overall								Pathology						
				Total: 85,16% recommended 34.52% for consideration” 50.64%								Squamous cell carcinoma: - Total: 79.12% - recommended 70.33% - for consideration 8.79%,						
												Adenocarcinoma: Total: 87.56% - recommended 19.82% - for consideration 67.74%						
Concordance Study Between IBM Watson for Oncology and Real Clinical Practice for Cervical Cancer Patients in China: A Retrospective Analysis	Zou FW et al.	Cervical	WFO	Overall					Stage			ECOG		Pathology	Metastasis			
				Total: 72,8% Recommended: 41,5% For consideration: 31,3%					Stage I: 41,4%			0-1: 77,9%		Squamous: 72,6%	Lymphatic-: 73,5% Lymphatic+: 71,9%			
									Stage II: 86,2%			2: 66,1%		Adeno: 75%	Distant-: 74,5% Distant+: 52,6%			
									Stage III: 86,7%			≥ 3: 23,1%		Adenosquamous: 70%				
									Stage IV: 7,6%					Small cell: 100%				
Early experience with Watson for Oncology: a clinical decision-support system for prostate cancer treatment recommendations	Yu SH et al.	Prostata	WFO	Overall:								Stage						
				Total: 73,6% Recommended: 53,2% For consideration: 20,4%								Stage I: 89,5%						
												Stage IIa: 87,1% Stage IIb: 70,3%						
												Stage III: 79,5%						
												Stage IV: 44,4%						
Concordance between treatment recommendations provided by IBM Watson for Oncology and a multidisciplinary tumor board for breast cancer in China	Zhao X et al.	Breast	WFO	Postoperative adjuvant treatment group								Metastatic group						
				Overall		Stage			Receptor	Menopausal		Overall		Receptor			Menopausal	
				Total: 77% Recommended: 57,5% For consideration: 19,5%		Stage I: 49,3%			HR+: 73,9%	Pre: 80,7%		Total: 27,5% Recommended: 17,7% For consideration: 9,8%		HR+: 31%			Pre: 26,9%	
						Stage II: 90,6%			HER2/neu+: 77,2%	Post: 72,9%				HER2/neu+: 25,9%			Post: 27,8%	
						Stage III: 92,6%			Triple-: 90,6%					Triple-: 18,8%				
Artificial intelligence and lung cancer treatment decision: agreement with recommendation of multidisciplinary tumor board	Kim MS et al.	Lung	WFO	Overall					NSCLC					SCLC				
				Total: 92,4%					Stage I: 90,3%					Limited: 94,9%				
									Stage II: 86,8%					Extensive: 100%				
									Stage III: 85,3%									
									Stage IV: 100%									
Effect of an Artificial Intelligence Clinical Decision Support System on Treatment Decisions for Complex Breast Cancer	Xu F et al.	Breast	WFO	- Ten oncologists provided blinded treatment recommendations before and after viewing therapeutic options offered by WFO. - analyses of treatment changes - Treatment decisions changed in 5% after reviewing WFO recommended treatment options patients - concentrated in those with hormone receptor (HR)–positive disease or stage IV disease (73% and 58%, respectively) - In 69% of the patient cases with decision changes, initial treatments were replaced, in the remaining 31% of patient cases, the recommended treatment was added as equivalent option for the patient to consider														
Concordance Between Watson for Oncology and Multidisciplinary Teams in Colorectal Cancer: Prognostic Implications and Predicting Concordance	Mao C et al.	Colorectal	WFO	Overall					Location					Stage				
				Total: 66,9% Recommended: 44% For consideration: 22,9%					Right Colon: 53,5%					Stage I: 87,5%				
									Left Colon: 68,4%					Stage II: 75,3%				
									Rectal: 72,6%					Stage III: 55,2%				
														Stage IV: 47,1%				
Comparison of an oncology clinical decision-support system's recommendations with actual treatment decisions	Suwanvecho S et al.	Breast, Colon, Lung, Rectal	WFO	By cancer type, all stages combined								By stage, all cancers combined						
				Breast: 59,3%								Stage I: 70%						
				Colon: 84,3%								Stage II: 73,1%						
				Lung: 68,2%								Stage III: 81,6%						
				Rectal: 86,2%								Stage IV: 63,2%						
Artificial Intelligence in Decision-Making for Colorectal Cancer Treatment Strategy: An Observational Study of Implementing Watson for Oncology in a 250-Case Cohort	Aikemu B et al.	Colorectal	WFO	Colorectal					Colon					Rectal				
				Overall: 91%					Overall: 91%					Overall: 91%				
				Stage II: 83%					Stage II: 94%					Stage II: 72%				
				Stage III: 94%					Stage III: 92%					Stage III: 93%				
				Stage IV: 88%					Stage IV: 88					Stage IV: 89%				
Adequacy and Effectiveness of Watson For Oncology in the Treatment of Thyroid Carcinoma	Yun HJ et al.	Thyroid	WFO	Overall: Recommended 48%; For Consideration 4%														
				Stage I: Recommended 52,4%; For Consideration 2,4%														
				Stage II: Recommended 50%; For Consideration 0%														
				Stage III: Recommended 16,7%; For Consideration 16,7%%														
Conversion of a colorectal cancer guideline into clinical decision trees with assessment of validity	Keikes L et al.	Colorectal	Decision tree system (software on Oncoguide)	- Overall. 81% - in 92% of non-concordant cases no guideline recommendation was available														
Machine-learning algorithm to predict multidisciplinary team treatment recommendations in the management of basal cell carcinoma	Andrew TW et al.	Basal cell	Decision tree model	- 37.5% of patients could be reliably predicted to be triaged to Mohs micrographic surgery (MMS), based on tumor location and age - choice of conventional treatment (surgical excision or radiotherapy) by the MDT could be reliably predicted based on the patient’s age, tumor phenotype and lesion size - the algorithm reliably predicted the MDT decision outcome of 45.1% of nasal Basal cell cancer														
Concordance assessment of Watson for Oncology in breast cancer chemotherapy: first China experience	Pan H	Breast	WFO	Overall: Total 69,41%; Recommended: 25,13%; For consideration: 44,27%														
				Adjuvant Chemotherapy								Neoadjuvant chemotherapy						
				Total: 65,03%								Total: 96,67%						
				Pathologic TMN Stage					Molecular subtype			Clinical TNM stage		Molecular subtype				
				Stage I: 50,76%					Luminal A: 55,29%			Stage II: 96,84%		Luminal A: 100%				
				Stage II: 64,93%					Luminal B/HER2-: 52,74%			Stage III: 95,45%		Luminal B/HER2-: 98,59%				
				Stage III: 92,13%					Luminal B/HER2+: 80,25%					Luminal B/HER2+: 95,12%				
									HER2+:77,86%					HER2+:96,77%				
									Triple-: 89,31%					Triple-: 93,10%				
Using guideline-based clinical decision support in oncological multidisciplinary team meetings: A prospective, multicenter concordance study	Ebben KCWJ et al.	Breast, Prostate, Colorectal	Clinical decision trees (Oncoguide)	Breast					Colorectal					Prostate				
				Total: 85,3%					Total: 88,9%					Total: 78.8%				
Watson for oncology decision system for treatment consistency study in breast cancer	Liu Y et al.	Breast	WFO	Postoperative								Advanced stage						
				Overall: 80,2%								Overall: 50,5%						
				Chemotherapy: 85,2%								Endocrine: 7%						
				Targeted Therapy: 89,2%								Chemotherapy: 43,5%						
				Radiation: 96,6%								Targeted+Endocrine Therapy: 20%						
				Endocrine: 99,6%														

## 4 Discussion

The objective of this review was to provide an overview and systematic analysis of the current research landscape, usage and accuracy of AI-based decision support systems in MDM. AI-based CDSS and MDM decisions have been evaluated according to consistency.

### 4.1 Limitation and disadvantages

While conducting a review on concordance, it was found that many studies from Asia were focused on the use of the WFO system. WFO was originally based on vast cancer treatment experience in North America and the National Comprehensive Cancer Network guidelines (14). Therefore, it is not surprising that there have been numerous studies on its concordance in other countries and this could affect the results and match rates. For example treatment recommendations for different types of cancer can differ significantly between countries, for example gastric cancer treatment in the US and Chinese population (48). Another example, while WFO recommends three immunotherapies, namely pembrolizumab, nivolumab, and atezolizumab, for metastatic NSCLC, these are not yet approved by the China Food and Drug Administration (CFDA) (21). Although WFO does not require all information, studies have shown that entering more data into the system could increase the concordance rate (20). It therefore also seems important to collect as much data from the population the system is used in.

When considering other CDSSs used, the studies available for analysis are limited, making it more challenging to draw conclusions about the consistency of treatment decisions compared to MDM.

Another significant issue is the variability in the definition of concordance. WFO overall concordance rate is often listed as *´Recommended´* and *´For consideration´* with these two categories sometimes being reported separately. It is crucial to carefully examine how the overall match is evaluated, as a high overall agreement may not always translate to a high “recommendation” but may only be viewed “for consideration”.

When treating cancer patients, an MDM is an integral part of treatment planning and approach (3, 49). Studies have already shown that oncology patients benefit from a multidisciplinary approach to health care (50–52). Therefore, discussion in an MDM should be considered fundamental in treatment decision-making. Consequently, the decisions of the CDSS should only be compared with the decisions of the MDM. In some studies, however, only a comparison between decisions regarding the actual treatment of patients and the CDSS was made (26–28, 30, 36, 38, 43). In part, even in some studies the CDSS decision was only compared to national guidance (17, 40). Moreover, the decisions of an MDM or actual treatment are not always consistent with the guidelines (53).

### 4.2 Concordance analyses

This review highlights a range of different tumor types with particular focus on breast, lung and colorectal cancer. These cancers are among the most frequently diagnosed worldwide (2), so it is understandable that more studies have been conducted on these types. The number of studies conducted for each cancer type allows for reasonable conclusions to be drawn about the agreement rate between the CDSS and MDM. However, for other tumor types, such as HCC, thyroid, prostate, cervical, ovarian and basal cell cancer only a few studies have been conducted, making it difficult to draw definitive conclusions. Among the various tumor types, breast cancer studies are the most consistent, with high agreement rates observed across different CDSS. These studies also tend to involve larger sample sizes, with most studies including more than 1000 patients compared to studies on other tumor types (16–18, 36, 43).

The review demonstrates a wide range of concordance rates across different studies, with some studies showing rates above 90% (19, 35, 39), while others are below 60% (29, 40). Therefore, it is crucial to only use a CDSS in clinical practice when there is a high concordance rate to ensure high confidence in decision-making. Breast cancer studies have shown the highest overall concordance rate, exceeding 90% in some studies (15, 16, 19) but still showing a wide range with even reported concordance rates below 60% (38). The concordance rates for gastric, thyroid and basal cell cancer are consistently the lowest. Regarding the agreement rates for individual stages, there is no general statement as it varies between studies and tumor types. The meta-analysis for different carcinomas showed no significant difference between stage I-II and stage III-IV (**Figure 3**). For breast cancer, however, there was a significant difference, so the concordance rate was higher at advanced stages. For colorectal carcinoma, the studies that performed a staging analysis also showed low concordant rates. Thus, it is important to note that some studies showed a high overall concordance rate when no differential stage analysis was performed.

However, a lower ECOG score seems to be associated with a higher concordance in the results. Furthermore, in studies comparing the treatment recommendation for NSCLC and SCLS, SCLC shows a higher concordance rate than NSCLC. In NSCLC, adenocarcinoma has a higher concordance rate than Squamous cell carcinoma.

### 4.3 Comparison to work done in this field

Jie et al. (14) published a meta-analysis on the application of WFO in 2021. However, only studies on WFO were included here (n = 9). Since then, multiple studies on AI in MDM have been published. The main purpose of the review by Jie et al. was to analyze the concordance rate between MDM and CDSS which was similar to our review. In comparison to our study only WFO was analyzed and less studies were included. One important difference was the concordance rate between the stages. The study by Jie et al. showed a higher agreement for lower stages, but without statistical significance, and a slightly higher overall agreement in comparison to our study. In our study, there was no significant difference in this regard, except for breast cancer. However, the subdivision was different. Thus, in contrast to us, Jie et al. subdivided into stages I-III and IV. Gastric cancer also showed the lowest agreement rates in Jie et al. A low ECOC also seemed to have been associated with a higher agreement rate in Jie et al. They also showed a higher consistency of SCLC compared to NSCLC, which was similar to our study.

### 4.4 Future perspective

In the near future, CDSS could be used in daily clinical routine. However, it is necessary to train the various systems based on large patient data sets. Moreover, verification of the accuracy of these data must take place on large patient collectives. The highest medical evidence is desirable and can be reached by conducting multicenter studies. This is certainly a major obstacle, since many hospitals use their own hospital information systems, making it more difficult to develop systems that can be used between different hospitals.

Should these systems prove to be highly accurate, then the use of CDSS in MDM can bring both a time saving and a qualitative gain. However, complete decision-making power by a CDSS should not be granted yet due to the importance and complexity of the decisions made during MDMs. However, it is conceivable that decision proposals are made by the CDSS and that the medical staff only has to approve them. Furthermore, the system should also recognize and indicate complex or individual cases and serve the latest scientific studies for the cases. Lastly, the automatic preparation of MDM cases is also a conceivable support for the medical staff.

## 5 Conclusion

This review and meta-analysis provides a basic overview of previous work in the field of AI and MDM. In particular, concordance rate between CDSSS and MDM was assessed and compared. WFO is certainly the most widely used system, especially in the USA and Asia. Therefore, there are currently the most studies and data on this system. The use of WFO already allows some conclusions to be made, while the results are very heterogeneous. Some tumors show higher concordance rates than others. For instance, breast and lung cancer exhibit higher concordance rates than gastric cancer when using CDSS, yet WFO does not appear to be utilized in Europe. However, promising alternatives such as OncoDoc2 and Oncoguide exist in this region. AI holds the potential to revolutionize hospital workflows and enhance diagnostics and therapies for patients. However, to fully realize these benefits, it is crucial to conduct further studies on the concordance between CDSS and MDM decisions.

This systematic review provides a comprehensive overview of the current state of research and indicates that the use of CDSS in clinical practice is feasible, but additional research is required to fully evaluate its potential impact.

## Author contributions

RO, SN and FK elaborated hypothesis, constructed the search algorithm, and performed the literature search systematically. RO wrote the manuscript. FK and SN critically revised the manuscript and interpreted the data. FK edited the revision of the manuscript. All the authors read and approved the final manuscript.
